# Retrograde cerebral perfusion reduces embolic and watershed lesions after acute type a aortic dissection repair with deep hypothermic circulatory arrest

**DOI:** 10.1186/s13019-024-02814-8

**Published:** 2024-05-29

**Authors:** Jacob Ede, Karl Teurneau-Hermansson, Birgitta Ramgren, Marion Moseby-Knappe, Daniel Oudin Åström, Mårten Larsson, Johan Sjögren, Per Wierup, Shahab Nozohoor, Igor Zindovic

**Affiliations:** 1 Department of Clinical Sciences Lund, Department of Cardiothoracic Surgery, Lund University, Skåne University Hospital, Lund, Sweden; 2 Department of Clinical Sciences Lund, Department of Radiology, Lund University, Skåne University Hospital, Lund, Sweden; 3 Department of Clinical Sciences Lund, Department of Neurology and Rehabilitation, Lund University, Skåne University Hospital, Lund, Sweden; 4 Division of Occupational and Environmental Medicine, Department of Laboratory Medicine, Lund University, Lund, Sweden; 5 Division of Sustainable Health, Department of Public Health and Clinical Medicine, Umeå University, Umeå, Sweden

**Keywords:** Aorta, Dissection, Retrograde cerebral perfusion, Stroke, Embolism, Watershed lesions

## Abstract

**Background:**

To assess whether retrograde cerebral perfusion reduces neurological injury and mortality in patients undergoing surgery for acute type A aortic dissection.

**Methods:**

Single-center, retrospective, observational study including all patients undergoing acute type A aortic dissection repair with deep hypothermic circulatory arrest between January 1998 and December 2022 with or without the adjunct of retrograde cerebral perfusion. 515 patients were included: 257 patients with hypothermic circulatory arrest only and 258 patients with hypothermic circulatory arrest and retrograde cerebral perfusion. The primary endpoints were clinical neurological injury, embolic lesions, and watershed lesions. Multivariable logistic regression was performed to identify independent predictors of the primary outcomes. Survival analysis was performed using Kaplan-Meier estimates.

**Results:**

Clinical neurological injury and embolic lesions were less frequent in patients with retrograde cerebral perfusion (20.2% vs. 28.4%, *p* = 0.041 and 13.7% vs. 23.4%, *p* = 0.010, respectively), but there was no significant difference in the occurrence of watershed lesions (3.0% vs. 6.1%, *p* = 0.156). However, after multivariable logistic regression, retrograde cerebral perfusion was associated with a significant reduction of clinical neurological injury (OR: 0.60; 95% CI 0.36–0.995, *p* = 0.049), embolic lesions (OR: 0.55; 95% CI 0.31–0.97, *p* = 0.041), and watershed lesions (OR: 0.25; 95%CI 0.07–0.80, *p* = 0.027). There was no significant difference in 30-day mortality (12.8% vs. 11.7%, *p =* ns) or long-term survival between groups.

**Conclusion:**

In this study, we showed that the addition of retrograde cerebral perfusion during hypothermic circulatory arrest in the setting of acute type A aortic dissection repair reduced the risk of clinical neurological injury, embolic lesions, and watershed lesions.

**Supplementary Information:**

The online version contains supplementary material available at 10.1186/s13019-024-02814-8.

## Introduction

Acute type A aortic dissection (ATAAD) is a lethal disease with high mortality if not treated immediately [1, 2]. Even with emergency surgery, mortality and morbidity are high [3–5]. Neurological complications after ATAAD and its associated surgery are common, with a reported incidence of stroke of 10–16% and coma of 3–9% which have a negative impact on both short- and long term mortality [3, 6–10].

The vast majority of ATAAD surgical repairs are performed with an open distal anastomosis [11]. During this procedure, deep hypothermia is induced to protect the cerebral parenchyma from ischemia as circulatory arrest is required for the completion of the distal anastomosis. As an adjunct to hypothermic circulatory arrest (HCA), retrograde cerebral perfusion (RCP) and antegrade cerebral perfusion (ACP) have been introduced, potentially reducing the risk of ischemic injuries, especially in cases with expected prolonged HCA duration [12–14].

After the first use of continuous flow RCP was described in 1990, it was widely adopted but lost popularity due to the increasing use of ACP [15, 16]. However, RCP is still frequently used and was employed in 22–27% of patients undergoing ATAAD repair in large ATAAD databases [10, 16]. Several recent reports, however, have shown improved neurological outcomes with RCP as an adjunct to HCA as well as a reduction of embolic lesions in comparison with ACP [10, 17, 18]. Previous studies have been limited by small study populations or a lack of details regarding the specific characteristics of the neurological injury, making it difficult to understand the causality behind the neuroprotective effect of RCP.

The aim of this study was to assess whether RCP has a neuroprotective effect in ATAAD repair and to compare the rate of neurological injury, using both clinical assessment and radiological findings, and mortality in patients undergoing surgery for ATAAD with HCA with and without the addition of RCP.

## Methods

### Study design

This study was a single-center, retrospective, observational study and included all patients who underwent ATAAD surgery with HCA between January 1998 and December 2022 at our institution. Patients undergoing surgery without HCA or surgery with HCA with the adjunct of ACP were excluded. Data was prospectively entered into our departmental surgical database, and additional data were collected by retrospective chart review and radiological assessment. The study was approved by the Ethical Review Authority, Stockholm, Sweden (ref. 2021 − 01185, April 23, 2021). Individual patient consent was waived.

### Outcomes and definitions

The primary outcome measures were clinical neurological injury, defined as either stroke or coma as defined below, embolic cerebral lesions, and watershed cerebral lesions diagnosed on computed tomography (CT) or magnetic resonance imaging (MRI). The secondary outcome measures were 30 day mortality and long term mortality. Stroke was defined as clinically verified focal neurological deficits with a symptom duration of more than 24 h regardless of radiological confirmation. Postoperative coma was defined as Glasgow coma scale (GCS) motor score < 6, 48 h after termination of pharmacological sedation. If neurological symptoms were present, a neurologist was consulted. Embolic lesions were defined as ischemic lesions spread in the cerebral parenchyma, and watershed infarctions were defined as ischemic lesions occurring at the border zones between major cerebral artery territories resulting from cerebral hypoperfusion determined by CT or MRI. The radiological assessment was performed by a senior consultant neuroradiologist. Hypotensive shock was defined as systolic blood pressure < 90 mmHg, clinical signs of hypotension, or the preoperative use of vasopressors or inotropes. Malperfusion was defined as clinical signs of end-organ ischemia, and cerebral malperfusion was defined as focal neurological deficit or altered state of consciousness prior to surgery.

### Surgical technique

The surgical technique used at our institution has previously been described in detail [19]. In summary, repair was performed using median sternotomy, cardiopulmonary bypass, and intermittent cardioplegic arrest. The cannulation site varied at the discretion of each surgeon. The resection and inspection of the aortic arch and the distal anastomosis were performed under hypothermic circulatory arrest, with or without the use of RCP. If RCP was used, the vena cava superior was cannulated separately and was snared proximal to the cannula before circulatory arrest. An RCP flow of 5-10 ml/kg/min was used with a maximum pressure of 25 mmHg measured using the central venous catheter. If a limited distal repair (ascending aortic or hemiarch repair) was feasible, this approach was favored at our institution, but the technique used depended on the location of the intimal tear and the extent of dissection. Aortic arch procedures entailed re-implantation of any supra-aortic branch. Aortic valve replacement or total root replacement was performed when the dissection involved the coronary ostia or aortic valve, or in the presence of an aortic root aneurysm. When required, the competence of the aortic valve was restored via subcommissural plication, commissural resuspension, or valvuloplasty. Concomitant procedures (e.g. coronary artery bypass) were performed when either preoperative angiogram showed significant coronary artery stenosis or a dissected coronary ostia that could not be repaired was identified.

### Statistical analysis

Categorical variables were presented as numbers and percentages. Continuous data were reported as median with interquartile range or mean ± standard deviation depending on the distribution of data. Chi-square test, Fisher’s exact test, two sample T-tests, and Mann-Whitney U-test were used for intergroup comparisons when appropriate.

The hypothesized association between RCP and defined outcome measures was analysed using binary logistic regression. We performed separate crude and adjusted regressions for each outcome. The multivariable model was performed with a priori adjustment for clinical variables known to carry significant risk of neurological injury, i.e., age, diabetes mellitus, coronary artery disease, previous cardiac surgery, presentation with syncope, cerebral malperfusion, hypotensive chock, intramural hematoma, and DeBakey type 1 dissection [6, 20, 21]. We also adjusted for an interaction term between RCP and cerebral malperfusion to account for the potential bias of using or not using RCP in the presence of cerebral malperfusion.

Furthermore, we performed sensitivity analyses by removing patients with preoperative cerebral malperfusion from the analyses. In addition, we performed analyses on a data set where missing values had been imputed by a random forest approach, and patients were matched by propensity score using nearest neighbour matching with a caliper of 0.2 on all preoperative variables.

To evaluate overall survival, we generated Kaplan-Meier curves, and groups were compared using the log-rank test. All statistical analyses were conducted using R version 4.3.2 (R foundation, Vienna, Austria) and SPSS version 29 (IBM, New York, NY, USA).

## Results

### Study population

**Fig. 1:**
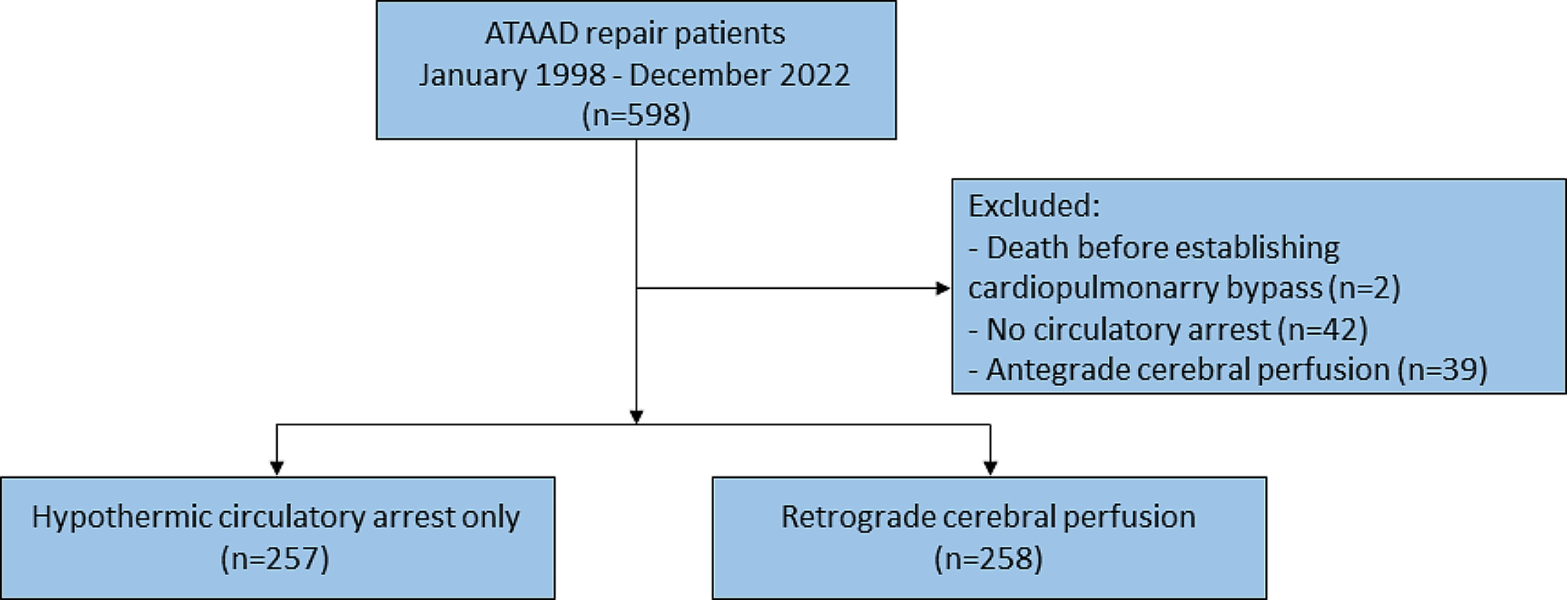
STROBE flow chart of patients in the study. Central image. The neuroprotective effect of retrograde cerebral perfusion compared to hypothermic circulatory arrest only

Between January 1998 and December 2022, 598 patients underwent ATAAD surgery at our institution. 515 patients underwent ATAAD surgery with HCA and were included in the present study. A total of 258 patients (50.1%) underwent repair with the use of RCP. In total, 83 patients were excluded: 42 because of surgery performed without circulatory arrest, 39 due to the use of ACP, and 2 patients because they died before the initiation of CPB (Fig. 1). Follow-up was conducted in March 2023 with a total of 3212 patient years (median 5.0 (1.1–10.1), mean 6.2 ± 5.8).

Baseline variables are presented in Table 1. A significantly lower proportion of patients in the RCP group presented with hypotensive shock (19.5% vs. 28.2%, *p* = 0.029), any malperfusion (30.7% vs. 40.5%, *p* = 0.027), and intramural hematoma (7.4% vs. 15.6%, *p* = 0.005). DeBakey type 1 dissections were more frequently observed in the RCP group compared to the HCA only group (78.4% vs. 86.0%, *p* = 0.034). There was a higher frequency of cerebral malperfusion in the HCA only group (17.6% vs. 11.3%, *p =* 0.057), but it did not reach the level of statistical significance.

**Table 1:** Baseline variables of the study population

	RCP (*n* = 258)	HCA only (*n* = 257)	*p*	Missing
Age	64.90 ± 11.7	64.6 ± 10.7	0.723	0
Female	93 (36.0)	97 (37.7)	0.758	0
Hypertension	121 (46.9)	142 (55.3)	0.071	0
Diabetes mellitus	46 (17.8)	34 (13.2)	0.187	0
COPD	14 ( 5.4)	14 ( 5.4)	1.000	0
Smoking	84 (33.5)	86 (35.7)	0.673	23 (4.5)
Coronary artery disease	21 ( 8.1)	29 (11.3)	0.291	0
Thoracic aneurysm	24 ( 9.3)	28 (10.9)	0.639	1 (0.2)
Marfan syndrome	12 ( 4.7)	9 ( 3.5)	0.662	0
Family history of dissection	8 ( 3.1)	13 ( 5.1)	0.368	0
Previous cardiac surgery	5 ( 1.9)	7 ( 2.7)	0.760	1 (0.2)
Previous aortic surgery	8 ( 3.1)	11 ( 4.3)	0.634	0
Preoperative creatinine (µmol/L)	87 (73–110)	90 (74–112)	0.392	21 (4.1)
Syncope	39 (15.2)	49 (19.1)	0.292	1 (0.2)
Hypotonic shock	50 (19.5)	71 (28.2)	0.029	7 (1.4)
Preoperative arrest	7 ( 2.7)	14 ( 5.4)	0.181	1 (0.2)
Malperfusion (any)	79 (30.7)	104 (40.5)	0.027	1 (0.2)
Cerebral malperfusion	29 (11.3)	45 (17.6)	0.057	2 (0.4)
Intramural hematoma	19 ( 7.4)	40 (15.6)	0.005	1 (0.2)
DeBakey Type 1	221 (86.0)	200 (78.4)	0.034	1 (0.2)

Values are presented as mean ± standard deviation, n (%), or median (interquartile range). HCA: hypothermic circulatory arrest, RCP: retrograde cerebral perfusion, COPD: chronic obstructive pulmonary disease

Intraoperative data are presented in Table 2. RCP patients had longer time on cardiopulmonary bypass (202 min; 95% CI 168–248 min vs. 168 min; 95% CI 134–213 min, *p* < 0.001), longer cross-clamp time (98 min; 95% CI 71–142 min vs. 67 min; 95% CI 50–106 min, *p* < 0.001) and longer HCA time (26 min; 95% CI 20–34 min vs. 18 min; 95% CI 14–23 min, *p* < 0.001). Patients with RCP were operated on at a lower HCA temperature (17.0 ℃; 95% CI 16.0–19.0 ℃ vs. 18.5 ℃; 95% CI 18.0–20.0 ℃, *p* > 0.001), and underwent more extensive proximal aortic repair.

**Table 2:** Intraoperative data of the study population

	RCP (*n* = 258)	HCA only (*n* = 257)	*p*-value	Missing
Cardiopulmonary bypass time (min)	202 (168–248)	168 (134–213)	< 0.001	0
Crossclamp time (min)	98 (71–142)	67 (50–106)	< 0.001	0
Hypothermic circulatory arrest time (min)	26 (20–34)	18 (14-23)	< 0.001	2 (0.4)
Hypothermic circulatory arrest temperature (°C)	17 (16-19)	19 (18-20)	< 0.001	3 (0.6)
Arterial cannulation			0.134	0
- Femoral artery	190 (73.6)	196 (76.3)		
- Axillary artery	2 (0.8)	6 (2.3)		
- Ascending aorta/Arch	65 (25.2)	51 (19.8)		
- Left ventricle	1 (0.4)	4 (1.6)		
Distal surgical technique			0.514	0
- Ascending aorta	209 (81.0)	218 (84.8)		
- Hemiarch procedure	36 (14.0)	29 (11.3)		
- Arch procedure	13 (5.0)	10 (3.9)		
Proximal surgical technique			0.012	0
- Supracoronary graft	174 (67.5)	207 (80.5)		
- Bentall procedure	62 (25.0)	41 (16.0)		
- Supracoronary graft + isolated AVR	4 (1.6)	3 (1.2)		
- Root replacement with aortic valve repair	17 (6.6)	6 (2.3)		
- Other/not completed	1 (0.4)	0 (0.0)		
Additional CABG	20 (7.8)	17 (6.6)	0.742	0

Values are presented as n (%) or median (interquartile range). HCA: hypothermic circulatory arrest, RCP: retrograde cerebral perfusion, AVR: aortic valve replacement, CABG: coronary artery bypass grafting

Postoperative data are presented in Table 3. There were no significant differences except for the RCP group receiving fewer units of platelets (4 units (2-5) vs. 4 units (2-6), *p* = 0.036), and neurological outcomes as presented below. Mortality was equal between the groups with an intraoperative mortality of 3.9% vs. 2.7%, 30-day mortality of 14.0% vs. 12.3%, and in-hospital mortality of 15.2% vs. 13.4% (*p* = ns) in the RCP group and HCA only group, respectively.

**Table 3:** Postoperative data of the study population

	RCP (*n* = 258)	HCA only (*n* = 257)	*p*-value	Missing
Peak creatinine (µmol/L)	132 (91–219)	118 (94–202)	0.479	36 (7.0)
Peak lactate (mmol/L)	2.8 (2.2–3.8)	3.1 (2.2–4.2)	0.183	225 (43.7)
Bleeding first 24 h (ml)	680 (450–920)	630 (435–855)	0.425	223 (43.4)
Peak CKMB (µg/L)	27 (17–52)	27 (18–54)	0.559	178 (34.6)
Packed red blood cells (units)	4 (2-8)	4 (2-7)	0.209	113 (21.9)
Plasma (units)	3 (0–8)	3 (0–6)	0.207	113 (21.9)
Platelets (units)	4 (2-5)	4 (2-6)	0.036	113 (21.9)
Fibrinogen (g)	4 (4-7)	6 (4-8)	0.193	222 (43.1)
rFVIIa	29 (31.2)	78 (39.0)	0.245	222 (43.1)
Reoperated for bleeding	41 (16.0)	35 (13.6)	0.522	2 (0.4)
Ventilator > 48 h	93 (39.9)	99 (39.8)	1.000	33 (6.4)
Renal replacement therapy	24 (9.9)	29 (11.6)	0.637	22 (4.3)
Postoperative MI	6 (4.5)	16 (15.7)	0.007	280 (54.4)
Multiple organ failure	3 (2.6)	6 ( 2.9)	1.000	192 37.3)
Clinical neurological injury	50 (20.2)	71 (28.4)	0.041	17 (3.3)
Stroke	36 (14.3)	45 (18.0)	0.349	17 (3.3)
Coma	14 (5.9)	26 (10.4)	0.067	17 (3.3)
Embolic lesions	32 (13.7)	57 (23.4)	0.010	38 (7.4)
Watershed lesions	7 (3.0)	15 (6.1)	0.156	38 (7.4)
Intraoperative death	10 (3.9)	7 (2.7)	0.628	0
30-day mortality	36 (14.0)	31 (12.3)	0.661	6 (1.2)
In-hospital mortality	39 (15.2)	34 (13.4)	0.652	4 (0.8)

Values are presented as n (%) or median (interquartile range). HCA: hypothermic circulatory arrest, RCP: retrograde cerebral perfusion, CKMB: creatine phosphokinase-MB, rFVIIa: recombinant factor VIIa and MI: myocardial infarction

**Fig. 2:**
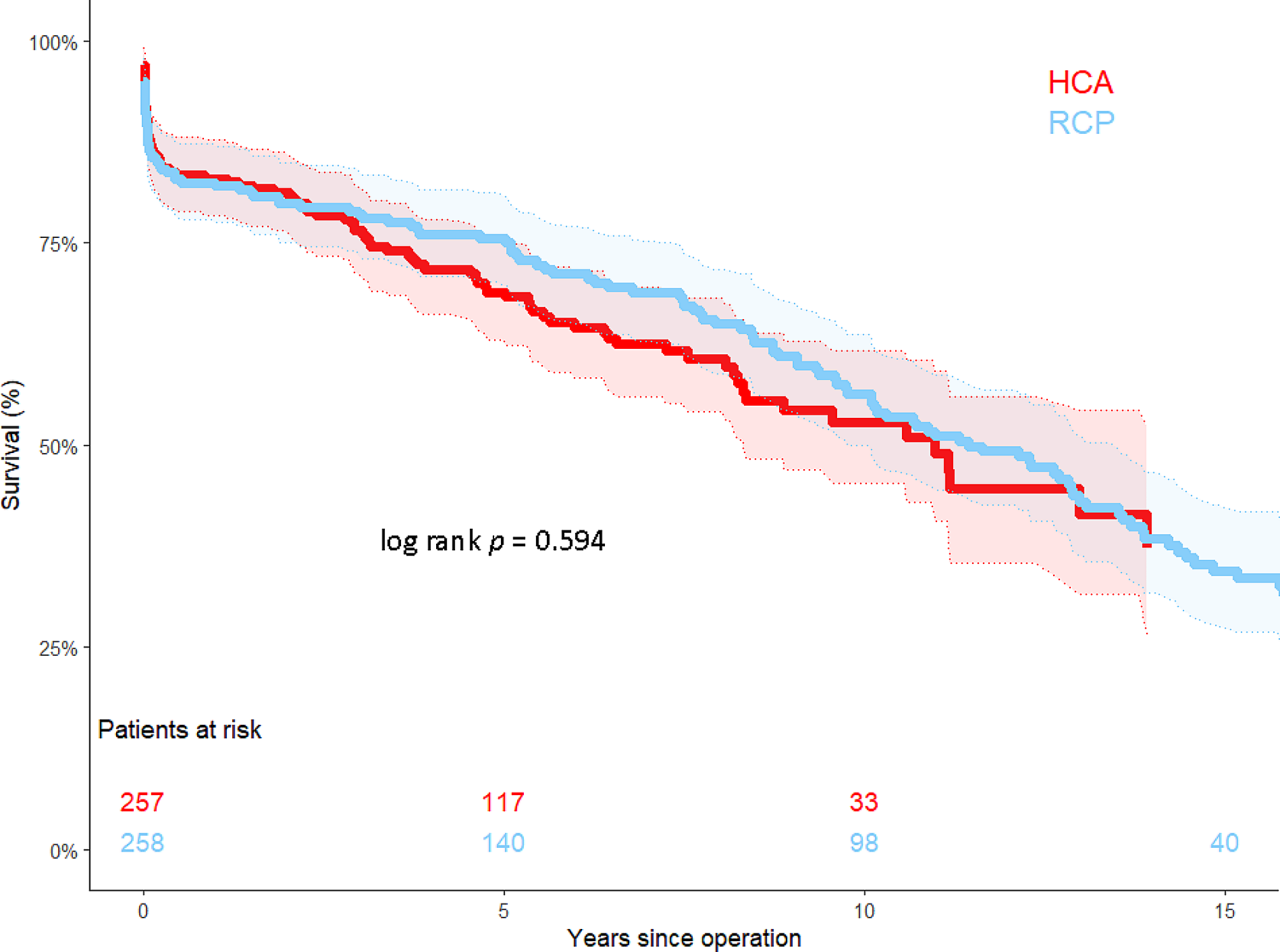
Kaplan-Meier survival curves of 30-day survivors demonstrating equal survival in both groups

Survival at 1, 5, and 15 years was similar for both groups with 82.0%±2.4% vs. 80.9%±2.4%, 73.9 ± 2.9% vs. 68.9%±3.1%, and 34.3 ± 3.8% vs. 37.2%±6.5% in the RCP and HCA only group, respectively (*p* = 0.594), Fig. 2).

### Neurological injury

Stroke or coma occurred postoperatively in 28.4% of HCA only patients and 20.2% of patients with RCP (*p* = 0.041) regardless of preoperative symptoms. In patients with a clinical neurological injury, 78.9% underwent a CT-scan of the brain, 12.4% underwent brain-MRI, and 16.5% underwent no radiological imaging. The median duration from surgery to imaging was 3 days (2-6). Embolic cerebral lesions were more frequently observed in the HCA only group (23.4% vs. 13.7%, *p* = 0.010). There was no significant difference in the occurrence of watershed lesions (6.1% vs. 3.0%, *p* = 0.156).

**Fig. 3:**
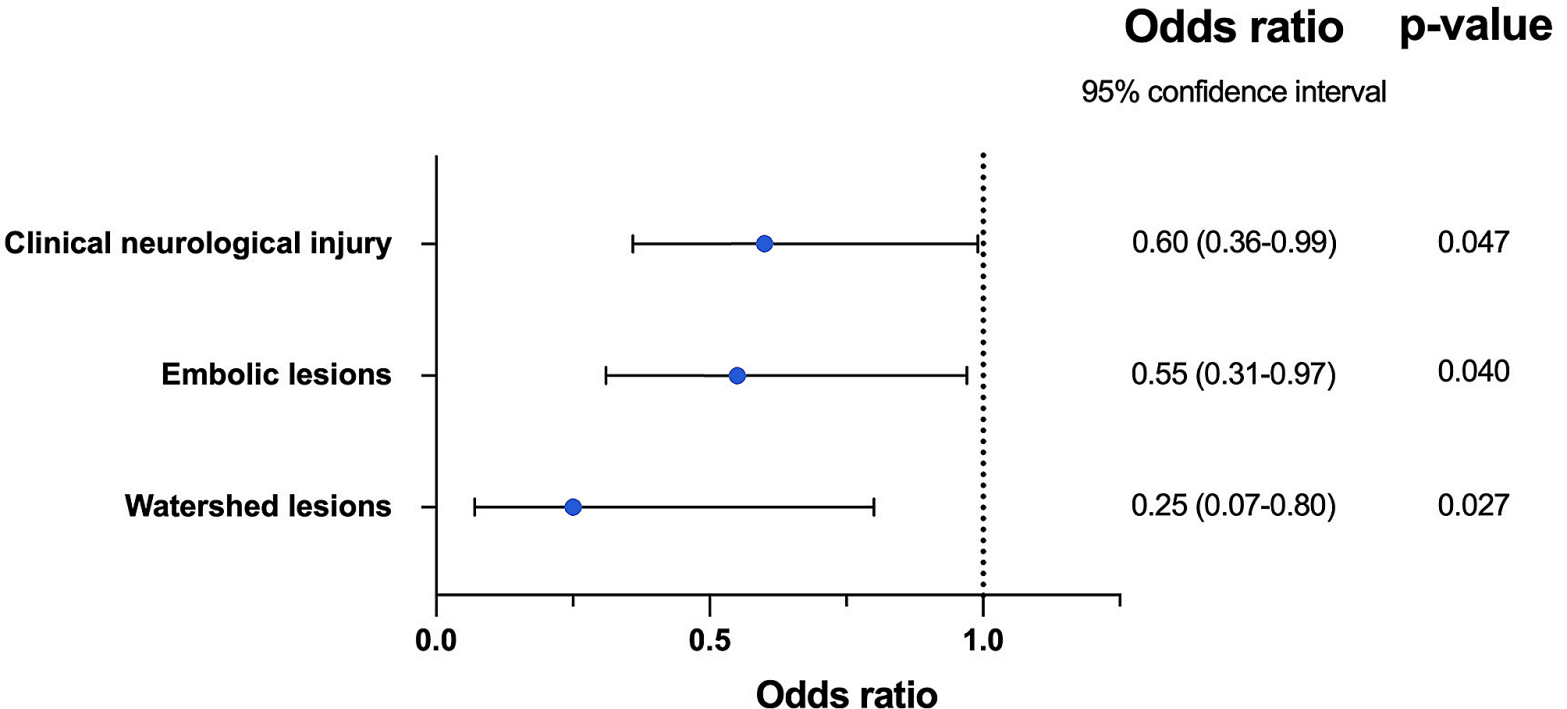
The neuroprotective effect of retrograde cerebral perfusion compared to hypothermic circulatory arrest only on the primary outcomes (clinical neurological injury, embolic lesions and watershed lesions)

After multivariable logistic regression with adjustment for covariates that may influence the risk of neurological injuries, the use of RCP independently predicted a significant reduction of clinical neurological injuries (OR: 0.60; 95% CI 0.36–0.99, *p* = 0.047), embolic lesions (OR: 0.55; 95% CI 0.31–0.97, *p* = 0.040), and watershed lesions (OR: 0.25; 95% CI 0.07–0.80, *p* = 0.027) (Fig. 3; Table 4).

**Table 4:** Crude and adjusted odds ratios for the primary outcomes in the full population and the population excluding patients with preoperative cerebral malperfusion

Full population					
Clinical neurological injury		Embolic lesions		Watershed lesions	
Crude	Adjusted	Crude	Adjusted	Crude	Adjusted
0.64 (0.42–0.96) *p* = 0.033	0.60 (0.36–0.99) *p* = 0.047	0.52 (0.32–0.83) *p* = 0.007	0.55 (0.31–0.97) *p* = 0.040	0.47 (0.18–1.14) *p* = 0.109	0.25 (0.07–0.80) *p* = 0.027
Study population without cerebral malperfusion					
Clinical neurological injury		Embolic lesions		Watershed lesions	
Crude	Adjusted	Crude	Adjusted	Crude	Adjusted
0.65 (0.40–1.06) *p* = 0.089	0.61 (0.36–1.02) *p* = 0.061	0.60 (0.34–1.04) *p* = 0.070	0.55 (0.31–0.98) *p* = 0.043	0.34 (0.09-1.00) *p* = 0.066	0.24 (0.06–0.77) *p* = 0.024

Values are presented as odds ratio (95% confidence interval). Adjusted analyses were adjusted for the following variables: age, diabetes mellitus, coronary artery disease, previous cardiac surgery, presentation with syncope, cerebral malperfusion, hypotensive chock, intramural hematoma, DeBakey type 1. The full population was also adjusted for an interaction term between RCP and cerebral malperfusion

In order to evaluate the robustness of our findings, we performed a series of sensitivity analyses. First, we excluded patients who presented with cerebral malperfusion. In the remaining cohort (*n* = 441), the use of RCP resulted in an adjusted OR of 0.61 (95% CI 0.36–1.02, *p* = 0.061) for clinical neurological injury, 0.55 (95% CI 0.31–0.98, *p* = 0.044) for embolic lesions, and 0.24 (95% CI 0.06–0.77, *p =* 0.024) for watershed lesions (Table 4). Second, we performed a propensity score matched analysis generating 218 matched pairs. The matched variables and absolute standardized differences of the propensity score matching are presented in Supplementary Fig. 1. Using this set of data, our analysis generated an OR of 0.82 (95% CI 0.53–1.34, *p* = 0.415) for clinical neurological injury, 0.68 (95% CI 0.40–1.15, *p* = 0.156) for embolic lesions, and 0.67 (95%CI 0.24–1.73, *p =* 0.413) for watershed lesions. Supplementary Table 1 illustrates the number of patients with cerebral malperfusion and neurological outcomes in each analysis, showing that both sensitivity analyses were performed on a study sample with a significant reduction of neurological outcomes, rendering them under-powered to detect some of the differences observed in the crude and adjusted primary analyses.

## Discussion

In our study, we could show that RCP reduced the risk of clinical neurological injury, embolic lesions, and watershed lesions diagnosed by CT or MRI, compared to HCA alone in the setting of ATAAD repair. Although improved neurological outcomes with RCP as an adjunct to HCA have been previously reported, there is lack of data in the characteristics of the neurological injury and the causality of the neuroprotective effect of RCP.

RCP was introduced by Mills and Ochner to treat iatrogenic air embolism in 1980 [22]. Lemole and colleagues were the first to describe its use in ATAAD in 1982 but administered it intermittently every 20 min during HCA rather than continuously [12]. The first use of continuous flow RCP was described by Ueda and colleagues in 1990 [15], but despite being used for more than 30 years, the exact mechanism of action of RCP is not fully understood. Proposed mechanisms include the washout of embolic debris, cooling of the brain parenchyma, and possibly a metabolic effect on the cerebral parenchyma.

Recently, several studies have reported that RCP has a protective effect compared to HCA only and ACP. A large study from the Society of Thoracic Surgeons Adult Cardiac Surgery Database, including more than 7000 ATAAD patients, showed that RCP reduced the risk of stroke compared to HCA only (OR 0.75; 95% CI 0.61–0.93, *p* = 0.008) and ACP (OR 0.75; 95% CI 0.61–0.93, *p* = 0.007) [10]. Furthermore, in a meta-analysis by Tian et al. comparing more than 5500 patients who underwent aortic surgery with HCA, HCA only was associated with increased mortality (OR 1.75; 95%CI 1.16–2.63, *p* = 0.007) and stroke (OR 1.50; 95% CI 1.07–2.10, *p* = 0.02) compared to surgery using RCP [18].

Neurological injury related to ATAAD repair may occur at several time points. Preoperatively, it may be due to dissection of the arch vessels and impairment of cerebral circulation or the release of thrombi from the false lumen via an entry- or re-entry tear. Intraoperatively, it may be due to hypoperfusion, thrombotic embolism associated with arterial cannulation, or embolism of air trapped during circulatory arrest. Postoperatively, there are numerous causes of neurological injury, two of which are hypoperfusion and the use of procoagulants.

In the present study, a reduced risk of watershed lesions was observed in patients who were operated on with the addition of RCP, despite these patients having longer duration of circulatory arrest, CPB, and cross-clamping. Previous studies on porcine models have demonstrated that during circulatory arrest with retrograde cerebral perfusion (blood dyed using an ink solution), most of the blood administered was returned to the heart via the inferior vena cava whereas only a small amount of blood was found in the innominate artery. The sinuses of the brain were filled with ink, but blood volume found in the cerebral capillaries after RCP was only 10% of that when using ACP [23]. It also has been suggested that the azygos system is a major route for blood from the RCP cannula, especially when the superior vena cava is valvulated [24]. Also, in a small randomized trial by Bonser et al. in patients who underwent arch surgery and either had HCA only or the addition of RCP, no difference in post-arrest oxygen extraction, glucose extraction, or jugular oxygen levels was observed [25]. In contrast to these findings, a small study using fluorescein in the administered blood during RCP showed that the fluorescein reached the retina. As the retina is a part of the central nervous system, it was concluded that the blood during RCP must reach the brain as well [26]. Furthermore, a porcine model including animals that underwent surgery with either ACP, RCP, HCA or HCA with the addition of ice-packs, improved behavioural outcomes and superior recovery EEG were observed for all groups compared to the HCA only group. While the volume of blood returning via the aortic arch during RCP was just 4% of the volume administered via ACP, RCP resulted in a superior oxygen extraction rate compared to ACP [27]. These data indicate that some of the blood provided by the RCP reaches the cerebral parenchyma, and although less than that provided by ACP, it might be sufficient to meet the metabolic requirements of the hypothermic brain. Since watershed injuries are the result of global cerebral hypoperfusion and the duration of circulatory arrest is a known risk factor for postoperative neurological injury and watershed lesions, our results indicate that that RCP may provide some metabolic effect, protecting the brain from global ischemic injury at relatively short circulatory arrest times at low temperatures [21].

Data in the present study also demonstrated that RCP reduces the risk of embolic cerebral lesions. In a small, randomized study on patients undergoing elective hemiarch repair under circulatory arrest, Leshnower et al. showed that the use of RCP resulted in a lower number of ischemic lesions and smaller volume of ischemic lesions on MRI than did ACP [17]. Previous studies have shown that ACP reduces the metabolic changes associated with HCA [27, 28]. Since ACP has been shown to provide a superior metabolic effect compared to RCP, one may speculate that the effect of RCP to some degree is related to the washout of air and other embolic material from the aortic arch branch vessels.

In the current study, we made several attempts to assess the robustness of our findings to ensure appropriate interpretation of the results, taking into account that patients with preoperative cerebral malperfusion were included in the main analyses. Cerebral malperfusion was more frequently observed in the HCA only patients rendering some discrepancy between the groups and constituting a significant bias as cerebral malperfusion is associated with a two- to four-fold increase in risk of neurological injury [21, 29]. Although the choice to use RCP relied on surgeon preference and not on any specific patient characteristics, one may speculate that patients with preoperative malperfusion are considered more critically ill and that the surgeon may be less inclined to perform time-consuming technical maneuvers, which would delay the timing of circulatory arrest. However, we believe that excluding patients with preoperative cerebral malperfusion from our initial analysis would not have been justifiable as the RCP may reverse the cause of cerebral malperfusion. Preoperative neurological symptoms may be caused by either embolism or dissection affecting the circulation of the carotid arteries and their branches. An occlusion resulting in circulatory compromise inevitably causes blood to remain static in the vessel, resulting in the formation of thrombi. By using RCP prior to reinstituting antegrade flow, existing blood clots may potentially be flushed out from the aortic branch vessels, and intimal flaps may be restored by retrograde blood flow. Therefore, we made multiple attempts to adjust for preoperative cerebral perfusion in our analyses. The use of RCP was adjusted for several confounding variables in the main analysis, including cerebral malperfusion and the interaction between RCP and cerebral malperfusion. In addition, we performed two separate sensitivity analyses. First by repeating the multivariable logistic regression on a cohort excluding patients with cerebral malperfusion and demonstrating that the protective effect of RCP remained in relation to both embolic and watershed lesions. Second, to eliminate baseline differences between groups, we performed a propensity score matched analysis. However, this analysis rendered a power reduction of the multivariable logistic regression, and perhaps more importantly, a skewed reduction of patients with cerebral malperfusion. While all patients with cerebral malperfusion in the RCP group remained in the matched analysis, the rate of cerebral malperfusion was reduced by 23% in the HCA only group. Thereby, the analysis excluded those patients that perhaps may benefit most from RCP. Therefore, it is our opinion that the crude analysis adjusted for several variables including cerebral malperfusion was the most accurate to assess our endpoints.

This study is limited by its retrospective design and by a database missing potentially significant variables. Furthermore, some patients who had clinical neurological injury did not undergo radiological examination of the brain (most often due to being terminally ill and radiology therefore being considered redundant) and could not be classified as having embolic or watershed lesions. Patients who did not have clinical neurological injury could still have small ischemic lesions that were missed as all patients did not undergo a postoperative CT-scan or MRI of the brain. There may also be some uncertainty as to the origin of the embolic lesions as these could also be the result of local atherosclerotic plaques or intimal flaps causing regional hypoperfusion instead of embolism. Since most patients underwent CT and not MRI, the timing of CT could have influenced the sensitivity for detecting ischaemic lesions.

## Conclusion

In the present study, we showed that RCP reduces the risk of clinical neurological injury, embolic lesions, and watershed lesions compared to HCA only. The reduced risk of embolic lesions suggests that the effect of RCP may partially be the consequence of washout of air and other embolic material, while the reduced risk of watershed lesions may be related to RCP having some metabolic effect on the cerebral parenchyma.

### Electronic supplementary material

Below is the link to the electronic supplementary material.

Supplementary Material 1

## Data Availability

The datasets analysed during the current study are not publicly available due to limitations in the ethical approval.

## Glossary of abbreviations

ATAAD: Acute type A aortic dissection
HCA: Hypothermic circulatory arrest
RCP: Retrograde cerebral perfusion
ACP: Antegrade cerebral perfusion
CT: Computer tomography
MRI: Magnetic resonance imaging
GCS: Glasgow coma scale
COPD: Chronic obstructive pulmonary disease
AVR: Aortic valve replacement
CABG: Coronary artery bypass grafting
CKMB: Creatine phosphokinase-MB
rFVIIa: Recombinant factor VIIa
MI: Myocardial infarction
