# Supplementary material for: Retrograde cerebral perfusion reduces embolic and watershed lesions after acute type a aortic dissection repair with deep hypothermic circulatory arrest
Source: J Cardiothorac Surg. 2024 May 29;19:302. doi: 10.1186/s13019-024-02814-8 (PMC11134620; doi:10.1186/s13019-024-02814-8)

Supplementary Table 1. Number of patients with cerebral malperfusion and neurological outcomes in each analysis.

| Population | Grouping | Clinical Neurological injury |
| --- | --- | --- |
| Full population | HCA only | 71 (28.4) |
|  | RCP | 50 (20.2) |
| Full population with cerebral | HCA only | 47 (23.0) |
| malperfusion excluded | RCP | 36 (16.4) |
| PS matched population | HCA only | 53 (24.6) |
|  | RCP | 46 (21.9) |
|  | | |
| Population | Grouping | Embolic lesions |
| Full population | HCA only | 57 (23.4) |
|  | RCP | 32 (13.7) |
| Full population with cerebral | HCA only | 37 (18.4) |
| malperfusion excluded | RCP | 25 (12.0) |
| PS matched population | HCA only | 42 (19.9) |
|  | RCP | 28 (14.4) |
|  | | |
| Population | Grouping | Watershed lesions |
| Full population | HCA only | 15 (6.1) |
|  | RCP | 7 (3.0) |
| Full population with cerebral | HCA only | 11 (5.5) |
| malperfusion excluded | RCP | 4 (1.9) |
| PS matched population | HCA only | 11 (5.2) |
|  | RCP | 7 (3.6) |
|  | | |
| Population | Grouping | Cerebral malperfusion |
| Full population | HCA only | 45 (17.6) |
|  | RCP | 29 (11.3) |
| PS matched population | HCA only | 30 (13.7) |
|  | RCP | 29 (13.2) |

Values are presented as n (%). HCA: hypothermic circulatory arrest, RCP: retrograde cerebral perfusion, PS: propensity score.

Supplementary Figure 1. The propensity score matching variables and their absolute standardized mean differences.


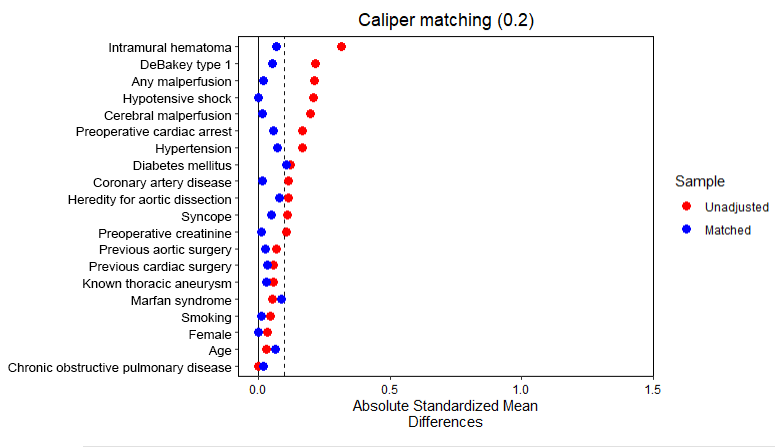

Supplement: Supplementary file 1 — Supplementary Material 1 [file 13019_2024_2814_MOESM1_ESM.docx]
